# Plastic Deformation of Micromachined Silicon Diaphragms with a Sealed Cavity at High Temperatures

**DOI:** 10.3390/s16020204

**Published:** 2016-02-05

**Authors:** Juan Ren, Michael Ward, Peter Kinnell, Russell Craddock, Xueyong Wei

**Affiliations:** 1 School of Mechanical Engineering, Xi’An Jiaotong University, 28 West Xianning Road, Xi’An 710049, China; seanwei@mail.xjtu.edu.cn; 2 Birmingham City University, Birmingham B5 5JU, UK; michael.ward@bcu.ac.uk; 3 Wolfson School of Mechanical, Electrical and Manufacturing Engineering, Loughborough University, Loughborough LE11 3UZ, UK; p.kinnell@lboro.ac.uk; 4 GE Sensing & Inspection Technologies, Leicestershire LE6 0FH, UK; russell.craddock@ge.com

**Keywords:** single crystal silicon, plastic deformation, pressure sensor, microfabrication

## Abstract

Single crystal silicon (SCS) diaphragms are widely used as pressure sensitive elements in micromachined pressure sensors. However, for harsh environments applications, pure silicon diaphragms are hardly used because of the deterioration of SCS in both electrical and mechanical properties. To survive at the elevated temperature, the silicon structures must work in combination with other advanced materials, such as silicon carbide (SiC) or silicon on insulator (SOI), for improved performance and reduced cost. Hence, in order to extend the operating temperatures of existing SCS microstructures, this work investigates the mechanical behavior of pressurized SCS diaphragms at high temperatures. A model was developed to predict the plastic deformation of SCS diaphragms and was verified by the experiments. The evolution of the deformation was obtained by studying the surface profiles at different anneal stages. The slow continuous deformation was considered as creep for the diaphragms with a radius of 2.5 mm at 600 °C. The occurrence of plastic deformation was successfully predicted by the model and was observed at the operating temperature of 800 °C and 900 °C, respectively.

## 1. Introduction

Single crystal silicon (SCS) is an excellent material for sensor applications because of its good mechanical properties, high purity and crystalline perfection [1]. For example, SCS diaphragms are widely used as pressure sensing elements for the micromachined pressure sensors. The magnitude of the pressure is normally determined by measuring the resultant strain or displacement of the diaphragm, which requires the diaphragms to work in the small deflection range under moderate pressure for a better linearity. It is well-known that SCS is a temperature-sensitive material and exhibits no plasticity or creep at normal temperature. Therefore, the conventional SCS-based sensors are free from hysteresis [2]. These diaphragms can also be shaped with high precision with the aid of the advanced silicon micromachining technology. With the rapid industrial development, high-temperature pressure sensors are demanded. However, traditional silicon sensors are incapable of working in high-temperature environments due to the deterioration in both electrical and mechanical properties. However, traditional silicon sensors are incapable of working in high-temperature environments due to the deterioration in both electrical and mechanical properties. For example, pure silicon sensors containing p-n junctions, for the electrical isolation cannot work properly at the temperature above 150 °C [3]. It is usually thought that conventional silicon-based electronics function at a temperature below 250 °C [4]. Meanwhile, silicon undergoes brittle-to-ductile transition at a temperature between 520 °C and 600 °C [5]. The stressed SCS components are therefore susceptible to creep at elevated temperatures. The continuous deformation may result in unacceptable dimensional change, as observed by Mehra *et al.* in a micromachined silicon combustor [6]. 

In order to be used in high temperature applications, silicon has been combined with other advanced materials, such as silicon carbide (SiC) or silicon on insulator (SOI). SiC exhibits good mechanical stability and stable electronic properties at temperatures up to 600 °C [7]. When the cost of a full SiC sensor is very high, it is advised that SiC components in SCS-based sensors are employed. Young *et al.* used the SiC diaphragm to form a capacitive pressure sensor [8]. Wu *et al.* implemented the SiC piezoresistors on silicon substrate [9]. The performances of these combined sensors were tested at temperatures up to approximately 400 °C. The SOI wafers contain an oxide layer between the device layer and the silicon substrate. Rather than using the p-n junctions, the SOI-based piezoresistive sensor designed by Guo *et al.* used the buried oxide layer to isolate the piezoresistors from the substrate and from each other [10]. The silicon diaphragm with carefully designed dimensions was used to sense the external pressure. The sensor showed a very low-pressure hysteresis up to 500 °C.

The mechanical behavior of the pressurized silicon diaphragm is partly dependent on the material properties of SCS. The yield strength in silicon has been studied by the conventional bending test and tensile test [11,12,13,14]. The creep properties for Si have previously been investigated using the uniaxial compression test and the four-point bending test [15,16,17,18]. When it comes to the silicon microstructures, however, it is found that the plastic behavior relates to the specimen size, the specimen orientation and the fabrication routes [19,20,21]. To optimize the fabrication using silicon wafer bonding, Huff *et al.* previously performed bulge test experiments on micromachined silicon membranes with the temperatures ranging from 900 °C to 1150 °C [22]. However, the creep effect in SCS microstructures has seldom been studied.

In order to extend the operating temperatures of existing SCS microstructures, this work investigates the mechanical behavior of pressurized SCS diaphragms at the temperature range of 600 °C–900 °C. Firstly, the design of the test samples and the fabrication process are presented, followed by the details of thermal treatment and profile measurement. Based on the reported critical resolved shear stress of silicon, the occurrence of the plastic deformation is then predicted using the orthotropic properties of silicon by the finite element method. After that, the maximum deflections for the diaphragms with radii from 0.5 mm to 2.5 mm are reported with respect to different annealing temperatures. The slip bands were observed on the surface of the plastically deformed diaphragm by the microscope. Finally, based on the evolution of the measured profiles, the size effect and the temperature effect on the diaphragm behavior are discussed. 

## 2. Experimental Details

### 2.1. Microfabrication of SCS Diaphragms with Sealed Cavity 

**Figure 1:**
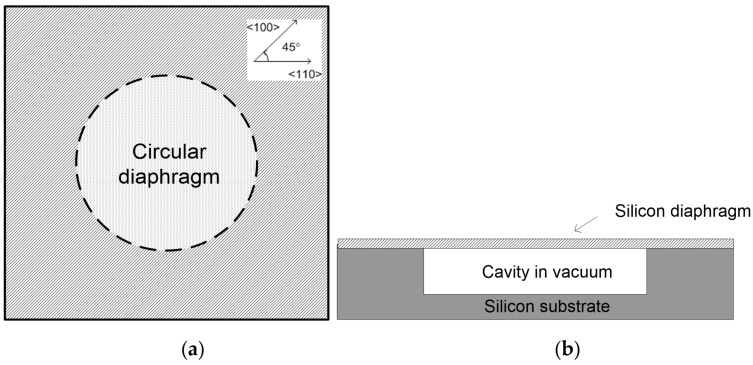
Schematic drawing of SCS diaphragm with sealed cavity (**a**) top view (inset is the crystal orientation of the device); (**b**) cross-section view.

The testing sample of the SCS diaphragm with a sealed cavity is illustrated in Figure 1, along with the crystal orientation of silicon. The silicon diaphragm is 50 µm thick and is bonded to the silicon substrate. The cavity with a depth of 300 µm under the diaphragm is sealed in a vacuum. The radius of the diaphragm is determined by the size of the cavity, and is in the range from 0.5 mm to 2.5 mm. The edges of the die are along <110> directions in the (100) plane.

**Figure 2:**
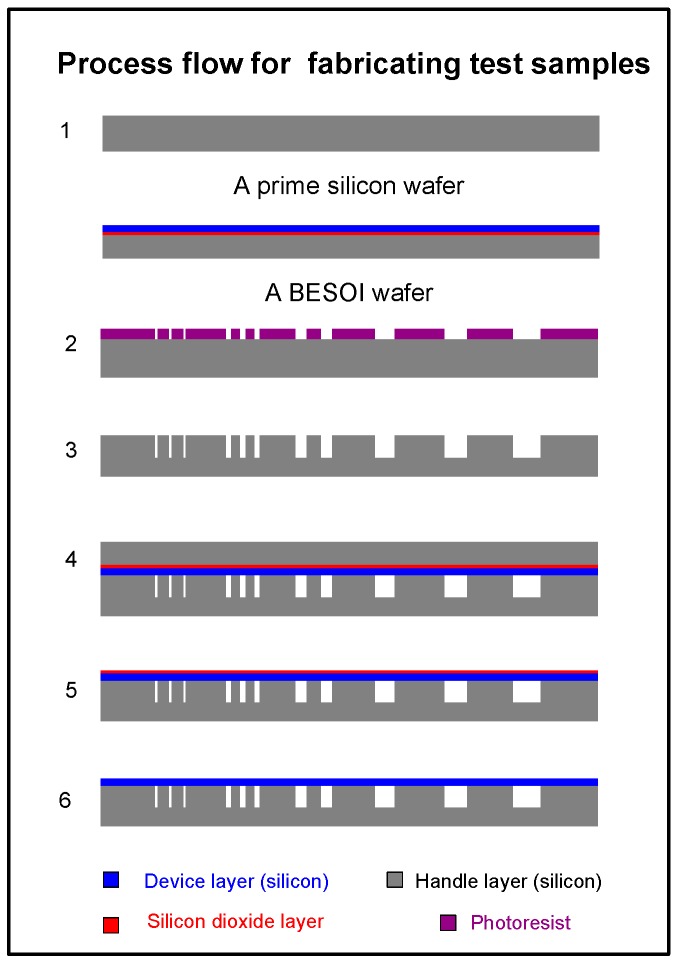
Process flow for fabricating SCS diaphragms with sealed cavity.

The fabrication of the testing samples has been reported in our previous work [23]. The process flow is briefly illustrated in Figure 2. The process starts with a 4-inch-diameter boron-doped <100> prime silicon wafer and a 4-inch-diameter <100> bond-and-etch-back-silicon-on-insulator (BESOI) wafer (step 1). The BESOI wafer has a 50 ± 0.5-µm thick single crystal silicon (SCS) device layer and a 0.5-µm ± 5% thick silicon dioxide layer on a 400 ± 5 µm thick SCS handle wafer. The SCS device layer is <100> oriented silicon with boron doping resistivity of 0.001–0.002 ohm-cm. Then, a thin layer of photoresist is deposited on the prime silicon wafer and patterned. In step 3, the front side of the patterned wafer is etched using the deep reactive-ion etching (DRIE). After that, the silicon substrate is bonded to a (100)-oriented BESOI wafer by the silicon fusion bonding method [24]. Then, the silicon handle layer of the BESOI wafer was completely etched away by the KOH wet-etching process (step 5). Finally, the samples were made ready for the subsequent tests after the wet etching of the silicon dioxide layer in an HF solution.

**Figure 3:**
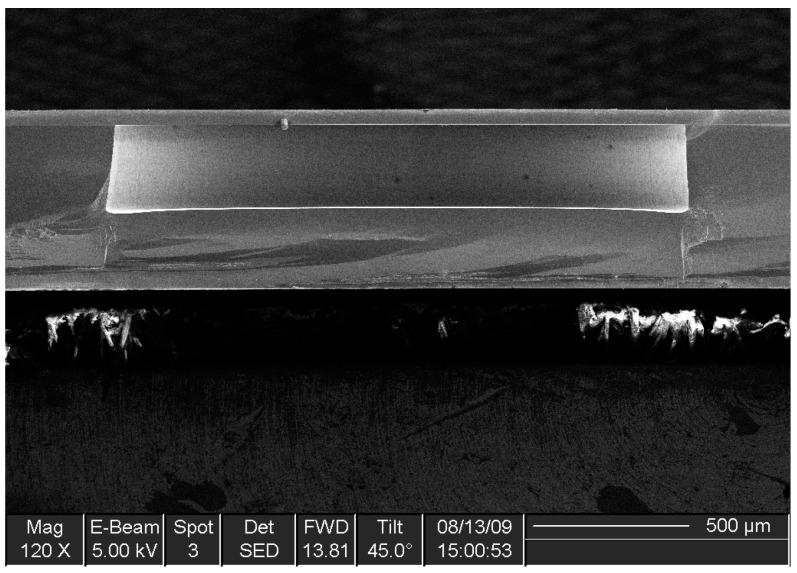
SEM image of a microfabricated SCS diaphragm (cross-sectional view).

Due to the pressure difference between the atmosphere and the sealed cavity, the silicon diaphragms deflected toward the substrate once they were made ready for test. The availability of the wet-etching method and the BESOI wafer allowed good control over the diaphragm thickness, as shown in Figure 3. The resulting diaphragm also had well-defined sidewalls. However, the manufacturing processes were not ideal. On the one hand, the boron dopant in silicon could have introduced internal stress in the diaphragms [25]. On the other hand, air may have been trapped inside the cavities, or the temperature for annealing may not have been evenly distributed during the wafer bonding process. The fabrication imperfections could have had an effect on the diaphragm behavior, and therefore need to be considered when the experimental data is evaluated. 

### 2.2. Thermal Treatment and Profile Measurement of SCS Diaphragms

The probable failure modes associated with the silicon microstructures at elevated temperatures are the plastic deformation or creep. The direct observation of the diaphragm deflection during annealing is experimentally very difficult. However, the brittle failure leads to the permanent change of the diaphragm deformation, and can be detected by studying the evolution of the surface profiles with annealing processes. Based on this concept, a series of experiments were conducted to investigate the size effect and the temperature effect on the deformation of the silicon diaphragms. 

The microfabricated SCS diaphragm was annealed in a furnace filled with nitrogen gas of atmospheric pressure for three different durations (namely Stage A, Stage B and Stage C) at each temperature tested in this study. In each annealing process, the furnace temperature rose from 20 °C with a ramp-up rate of 5 °C/min. Then, it was maintained at the annealing temperature for a duration specified in Table 1. The annealing duration was kept short in Stage A so that deformation due to plasticity could be observed. Based on the testing results after annealing Stage A, the annealing durations for Stage B and C were carefully designed in accordance with the operating temperature. By assuming that the creep rate is positively related to the annealing temperature, the annealing time at lower temperature was kept longer so that the deformation caused by creep could be measured. After that, the furnace was cooled down gradually to 20 °C with a ramp-down rate of −5 °C/min.

**Table 1:** The list of anneal time and temperature.

Anneal Temperature	Anneal Time (hour)		
	Stage A	Stage B	Stage C
600 °C	1	68	68
800 °C	1	15	15
900 °C	1	10	10

The surface profiles of the test samples were measured with a white light interferometer. All the measurements were taken under the atmospheric pressure at a temperature of 20 °C. At room temperature, silicon is brittle and the Young’s modulus is high. Elastic deformation was measured before annealing Stage A, and the magnitude was proportional to the atmospheric pressure. After annealing, as the samples cooled down to the room temperature, the silicon became brittle again. If the diaphragm deflected plastically during annealing, the measured data afterwards would consist of the plastic deformation and the atmosphere induced elastic deformation. Therefore, the high temperature behavior of the diaphragm can be determined by comparing its surface profile tested before and after the different annealing stages. 

## 3. Theoretical Prediction of Plastic Deformation 

### 3.1. Resolved Shear Stress in SCS

The plastic deformation in the single crystal silicon was caused by the dislocation slip, which was initiated by the shearing stress resolved on the slip plane in the slip direction. Wherever the acting resolved shear stress obtained from the operating temperature field exceeded the critical value (the yield strength), the crystallographic slip began. Because of the face-centered-cubic structure of silicon, the dislocations glided along <011> directions on the dense atomic planes {111}. There are a total of 12 primary slip systems for SCS. For each slip system s, the crystallographic orientation of the slip plane n^s^ and the slip direction l^s^ are listed in Table 2.

**Table 2:** The primary slip systems of single crystal silicon [26].

s	1	2	3	4	5	6	7	8	9	10	11	12
n^s^	111	111	111	$1\bar{1}1$	$1\bar{1}1$	$1\bar{1}1$	$\bar{1}11$	$\bar{1}11$	$\bar{1}11$	$11\bar{1}$	$11\bar{1}$	$11\bar{1}$
l^s^	$\bar{1}01$	$0\bar{1}1$	$\bar{1}10$	$\bar{1}01$	011	110	$0\bar{1}1$	110	101	$\bar{1}10$	101	011

The resolved shear stress on a slip system s is the scalar product between the orientation tensor, *m^s^*, and the macroscopic stress tensor, σ [27]:

(1) $$\tau^{s}=m^{s}:\sigma$$

where *m^s^* is given by:

(2) $$m_{ij}^{s}=\frac{1}{2}(n_{i}^{s}l_{j}^{s}+n_{j}^{s}l_{i}^{s})$$

### 3.2. FEA Modeling 

The pressure-induced stress in the test samples can be calculated by the aid of COMSOL Multiphysics 3.5a in 3-dimensional domain. Since the test samples were free to expand during high temperature annealing, there was no thermally induced stress. At the same time, the temperature dependence of the elastic properties of silicon was ignored in the modeling because its effect on the stress field is very small compared to the effect of the applied pressure. The resolved shear stress for all the slip systems could then be obtained by applying the Cauchy stress.

**Figure 4:**
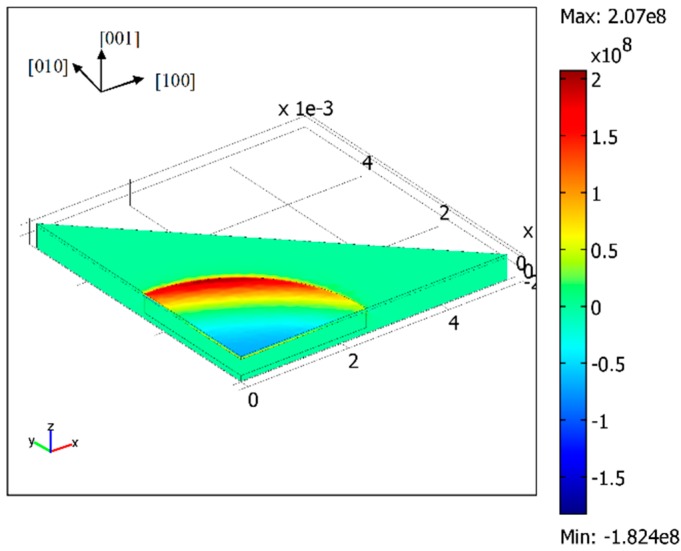
The distribution of the resolved shear stress for slip system 12 when the diaphragm radius is 2.5 mm (Pa).

Due to the orthotropic properties of SCS, the stress field of one slip system is different from that of the other [28]. It was found that the maximum resolved shear stress occurred at the edge of the diaphragm for the slip system 12, as shown in Figure 4. Because the test structure had two planes of symmetry, only a quarter of the geometry is modeled. The external pressure was applied on the upper surface of the sample. The bottom and the substrate sidewall were rigidly fixed. Since the definition of the resolved shear stress refers to the [100] crystal orientation, the x-axis of the model coordinate system aligns with crystallographic direction <100> in the (100) wafer plane. This model ignores the temperature dependence of the stiffness coefficients. The simulated stress is therefore not related to the operating temperature. Therefore, it is assumed that the maximum resolved shear stress in the test structure remained the same at elevated temperatures.

### 3.3. Occurrence of Plastic Deformation 

**Figure 5:**
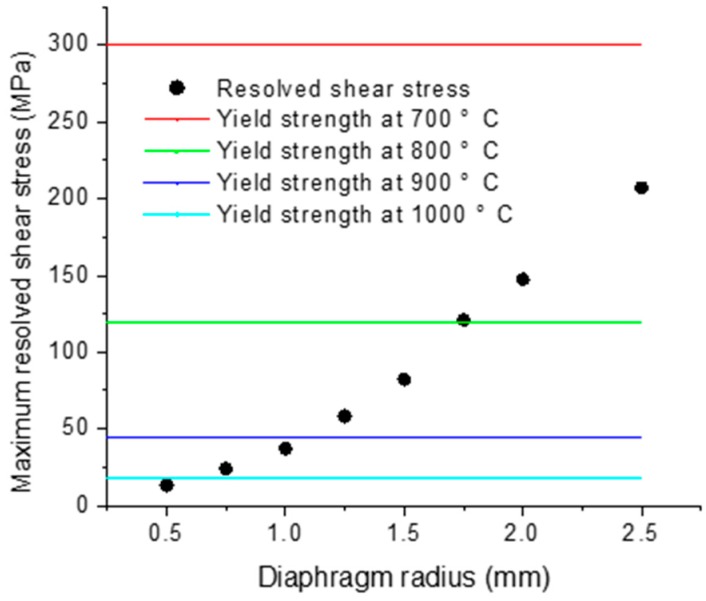
Plot of maximum resolved shear stress in diaphragm as a function of diaphragm radius (comparison of resolved shear stress with yield strength).

The slip of the dislocation began when the resolved shear stress reached the yield strength at the operating temperature. The onset of the plastic deformation could then be predicted simply by comparing the maximum resolved shear stress with the yield strength at each operating temperature, as shown in Figure 5. Here in this analysis, the yield strength for the dislocation-free silicon was used with a strain rate of 5 × 10^−3^ cm/min [12]. It can be seen that the maximum resolved shear stresses varied from 13 MPa to 207 MPa, well below the yield strength of 300 MPa at 700 °C. Consequently, all of the micromachined diaphragms would not fail by plastic deformation. At 800 °C, the yield strength decreases to about 120 MPa. The plastic deformation would have happened for the diaphragms with a radius from 1.75 mm to 2.5 mm. At 900 °C, the diaphragms with a radius in the range of 1.2–2.5 mm are very likely to deform plastically. When the operating temperature increases to 1000 °C, the plastic deformation can be serious for the diaphragms with a radius larger than 0.5 mm. The results suggest that the higher the operating temperature and the larger the diaphragm radius, the more likely the plastic deformation is.

### 3.4. The Plastic Zone

**Figure 6:**
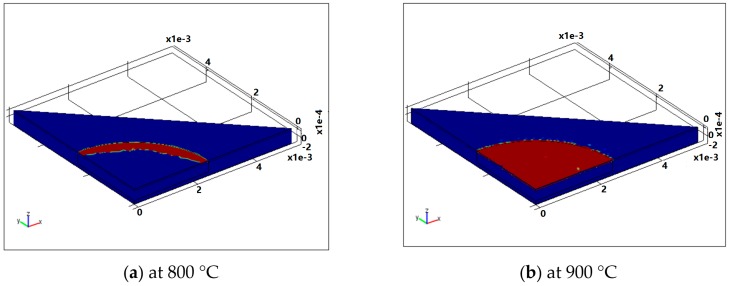
Plastic zones in the diaphragm at temperature of (**a**) 800 °C and (**b**) 900 °C with a radius of 2.5 mm (illustrated by red color) predicted by FEA model.

The region where the plastic deformation is likely to take place can also be predicted by the FEA model, as highlighted by the red color in Figure 6. It can be seen that, at 800 °C, the slip of dislocations might occur at the region close to the diaphragm edge. When the annealing temperature increases to 900 °C, almost the entire region of the diaphragm seems to be affected by the plastic deformation. Therefore, it is expected that the resultant deformation is more obvious at 900 °C than that at 800 °C.

## 4. Experimental Results and Discussion

### 4.1. Plastic Deformation and Creep at High Temperature

For the pressurized SCS diaphragms annealed at 600 °C, the evolution of their averaged maximum deflections at different annealing stage is recorded in Table 3. It can be seen that, when the radius is 2 mm and under, there is no measurable differences among the deflections. Therefore, the deformation is considered elastic, and the effect of creep can be ignored. 

**Table 3:** The averaged maximum deflections (μm) with respect to the diaphragm radius at 600 °C.

	Total Annealing Time	0.5 mm	0.75 mm	1 mm	1.25 mm	1.5 mm	1.75 mm	2 mm	2.5 mm
Before annealing	0 h	0.094	0.381	1.247	2.842	5.803	10.706	17.366	36.718
After annealing A	1 h	0.097	0.398	1.226	2.841	5.804	10.756	17.542	37.457
After annealing B	69 h	0.095	0.388	1.205	2.822	5.780	10.681	17.833	61.817
After annealing C	137 h	0.090	0.390	1.208	2.811	5.775	10.621	17.990	74.780

**Figure 7:**
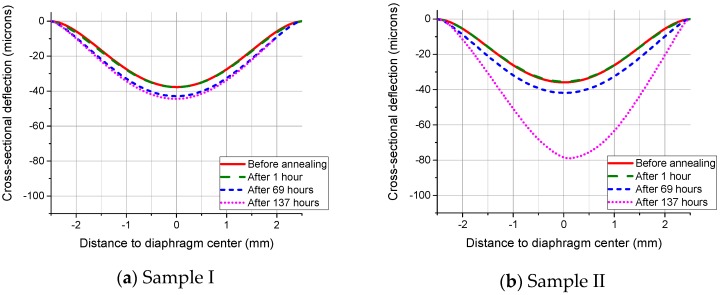
Surface profiles of SCS diaphragm cross-sections with a radius of 2.5 mm measured after the annealing processes at 600 °C, showing a different behavior.

For the diaphragms with the radius of 2.5 mm, they showed very different behavior, as indicated by the two samples’ surface profiles shown in Figure 7. For silicon, 600 °C is exactly the threshold temperature of creep. Because there were no apparent changes for the deflections in the anneal Stage A, the noticeable deformation increases afterwards are contributed by the mechanism of creep. Since the elastic deflections before annealing are quite similar, it is supposed that the volumes of the air sealed in the cavities are quite the same. Meanwhile, these two samples are located close to the wafer center. Therefore, any structural differences caused by the fabrication imperfections or any slight variations in temperature could either speed up or slow down the creep process.

**Figure 8:**
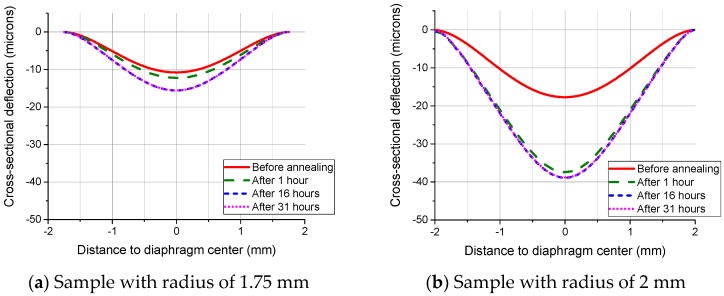
Surface profiles of SCS diaphragm cross-sections with a radius of (**a**) 1.75 mm and (**b**) 2 mm measured after the annealing processes at 800 °C.

Table 4 shows the averaged maximum deflections of pressurized SCS diaphragms annealed at 800 °C. When the radius is 1.5 mm or under, there are no measurable differences among the deflections. It is the elastic deformation that happened during annealing. However, the deflection becomes larger when the radius increases to 1.75 mm or larger, as shown in Figure 8. It can be seen that the deflections induced in the anneal Stage A are quite obvious. Therefore, it is the plastic deformation that dominates in the diaphragm behavior. The data also shows that there is no measurable creep in the anneal Stage C. 

**Table 4:** The averaged maximum deflections (μm) with respect to the diaphragm radius at 800 °C

	Total Annealing Time	0.5 mm	0.75 mm	1 mm	1.25 mm	1.5 mm	1.75 mm	2 mm	2.5 mm
Before annealing	0 h	0.087	0.375	1.149	2.895	5.902	10.790	17.517	37.090
After annealing A	1 h	0.086	0.378	1.143	2.866	6.001	11.926	37.902	109.251
After annealing B	16 h	0.084	0.383	1.156	2.877	6.027	13.491	38.518	110.096
After annealing C	31 h	0.088	0.386	1.164	2.886	6.056	13.532	38.507	110.950

**Figure 9:**
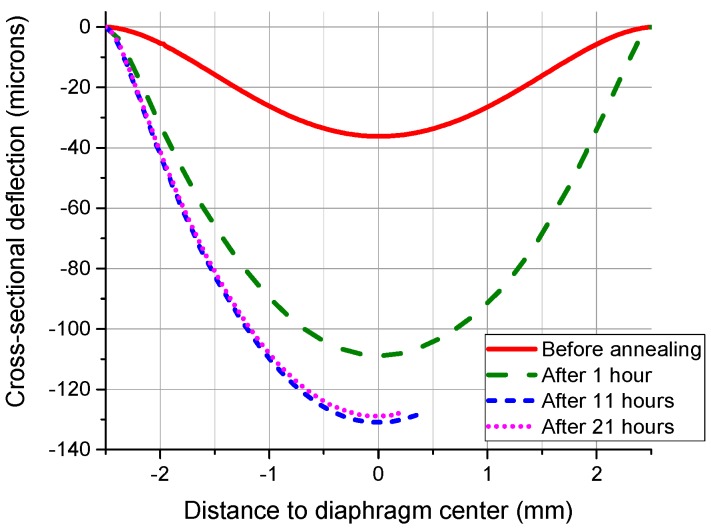
Surface profiles of SCS diaphragm cross-sections with a radius of 2.5 mm measured after the annealing processes at 900 °C.

When the annealing temperature was increased to 900 °C, neither plastic deformation nor creep effect was evident when the diaphragm radius was 1 mm or smaller, as indicated in Table 5. For others, the plastic deformation is quite noticeable in the anneal Stage A and B. It should be noted that, in the anneal Stage C, negative creep took place for the diaphragms with a radius of 2 mm and 2.5 mm, as shown in Figure 9. Negative creep has been previously observed during the bending of the boron-doped silicon [29]. The suggested mechanism to account for this phenomenon is the redistribution of the vacancies and impurities in the transverse field of mechanical stress. In this case, the effect of the negative creep should not be ignored. When the diaphragm radius increases to 2 mm and 2.5 mm, the elastic deformation under atmosphere is large. The middle surface is appreciably strained in order to resist the lateral load. Therefore, when the thermal energy was high enough at 900 °C, the vacancies and the impurities redistributed under the transverse stress during anneal. As a result, the vertical displacement decreased. 

**Table 5:** The averaged maximum deflections (μm) with respect to the diaphragm radius at 900 °C.

	Total Annealing Time	0.5 mm	0.75 mm	1 mm	1.25 mm	1.5 mm	1.75 mm	2 mm	2.5 mm
Before annealing	0 h	0.091	0.394	1.164	2.884	5.887	10.755	17.308	37.211
After annealing A	1 h	0.092	0.403	1.172	2.936	6.342	17.549	39.135	110.107
After annealing B	11 h	0.089	0.400	1.176	3.192	10.665	35.278	86.778	134.680
After annealing C	21 h	0.085	0.416	1.190	3.220	10.749	35.449	85.980	130.516

**Figure 10:**
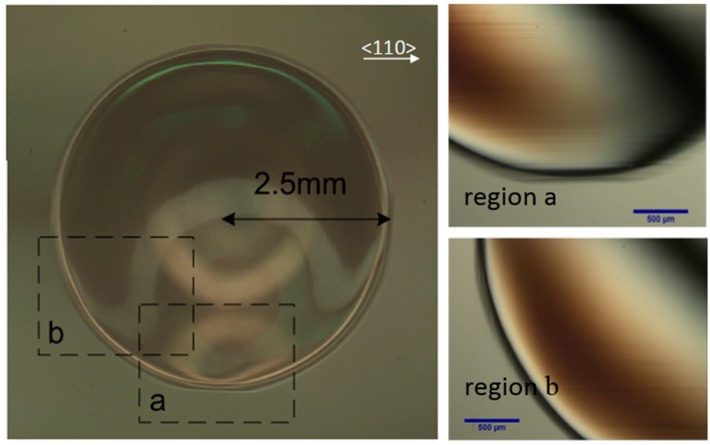
Top view optical microscope photo of diaphragm with a radius of 2.5 mm after annealing 900 °C for 21 h.

For the diaphragm presented in Figure 9, the microscopic photo of the deformed surface after annealing for 21 h is shown in Figure 10. The slip bands appeared as bright and dark lines as shown in Figure 2 and Figure 3. In region a, a high density of the visible lines are oriented parallel to the [110] direction, while, in the region b, the visible lines are oriented parallel and perpendicular to the [110] direction. The discrepancy in the arrangement of the slip bands is caused by the different number of working slip systems. Because silicon is orthotropic, the induced stresses vary from place to place. The photos suggest that the induced stress activates more slip systems at the specimen corner (region b) than the side (region a).

### 4.2. Discussion

The experimental results reveal that larger diaphragms have a higher risk of plastic deformation. Once the pressure applied on the test sample is determined, the larger the diaphragm radius, and the higher the stresses are induced. As a result, the slip of the dislocations is more likely to be activated once the critical value is achieved. 

Meanwhile, high temperature adds to the risk of elastic failure. When it comes to creep, raising temperature increases the diffusion rate of the silicon atoms in the pressurized diaphragms and therefore speeds up the creep progress. For plastic deformation, raising temperature decreases the value of the critical resolved shear stress (CRSS). Therefore, the pressurized diaphragms are more likely to deform plastically in a high-temperature environment. The higher the operating temperature, the larger the plastic deformation is. For the diaphragms with a radius of 2.5 mm, the maximum deformation is about 101 µm after annealing at 600 °C for 137 h, while it is about 112 µm after annealing at 800 °C for 31 h, and is about 135 µm after annealing at 900 °C for 21 h. The creep at 600 °C has a low deformation rate. 

**Figure 11:**
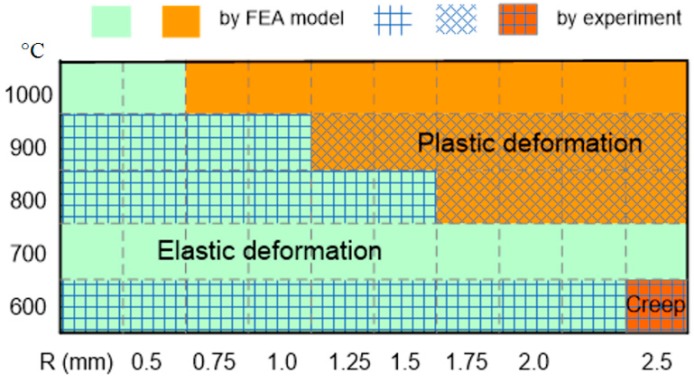
Evaluation of the prediction using the experimental observation.

The experimental results and the FEA prediction of the plastic deformation in the silicon diaphragms are compared and illustrated in Figure 11. The mechanical behavior is summarized as a function of the diaphragm radius and the operating temperature. It indicates that the predicted behavior is in good agreement with the experimental observation at 600 °C, 800 °C and 900 °C. Furthermore, this model is successful in predicting the plastic zone. However, a quantative approximation of the plastic deflection is complicated. 

## 5. Conclusions

Because the plastic deformation is a very complicated process, this work is focused on the mechanical aspect of the phenomenon. A series of experiments was performed to investigate the effect of size and temperature on the deformation of the silicon microstructures. The samples for testing were fabricated by the micromachining technology with good control over diaphragm thickness. By comparing the surface profiles obtained before and after a number of annealing processes, the trend of the diaphragm deformation is obtained. The results are fairly good for engineering applications.

The occurrence of the plastic deformation was predicted by comparing the resolved shear stress induced by pressure with the yield strength of SCS, which is a valuable criterion for predicting the elastic failure of SCS microstructures in high temperature applications. The prediction was verified by the experimental results. The accuracy of the FEA modelling can be improved by including the exact material properties and experimental conditions. When the plastic deformation can be avoided in the primary design, the creep deformation must be tested if the device is to operate at a temperature over 600 °C for a long period of time. 

The experimental results provide good reference data for designing the sensing diaphragm for high-temperature pressure sensors. The profile data imply that either increasing the size or raising the temperature adds to the risk of creep and plastic deformation. The diaphragms with smaller dimensions are more likely to survive in high-temperature environments. However, reducing the radius decreases the sensitivity of the diaphragm to pressure. Therefore, the dimension should be decided so that the device lifetime and sensitivity are well balanced. At the same time, the transduction mechanism needs to be chosen carefully so that the signal change caused by the external pressure can be sensed with a desired resolution.
